# Haptic and cognitive trade-offs of virtual reality–supported anatomy education: a retrospective multimethod cohort comparison

**DOI:** 10.1186/s12909-026-10481-7

**Published:** 2026-09-25

**Authors:** Songyuan Yao, Rui Liu, Xiju He

**Affiliations:** School of Basic Medical Sciences, Hubei University of Medicine, 30 Renmin South Road, Maojian District, Shiyan, Hubei Province 442000 China

**Keywords:** Anatomy education, Virtual reality, Cadaver-based learning, Haptic transfer, Knowledge retention, Educational technology, Cohort study

## Abstract

**Background:**

Immersive virtual reality (VR) can facilitate three-dimensional anatomy learning but may not reproduce the haptic information, representational variability, and retrieval demands encountered in physical anatomy learning. We examined performance beyond immediate recognition of standardized representations.

**Methods:**

We integrated data from three routine educational-assessment modules at one medical school, with the comparative analysis framework specified post hoc. Forty first-year students without previous cadaver-dissection experience received pure-VR or cadaver-based instruction in two intact classes (20 per condition). The three focal outcomes, designated post hoc for the integrated analysis, were peak force during a standardized cadaver task, accuracy for noncanonical CT/MRI cases, and the condition-by-time interaction in knowledge scores from 30 min to four weeks. Estimates are reported with 95% confidence intervals (CIs) and effect sizes.

**Results:**

The pure-VR condition showed greater peak force during transfer to cadaveric tissue (8.74 ± 2.31 vs. 4.52 ± 1.68 N; mean difference [MD], 4.22 N; 95% CI, 2.92–5.52; Cohen’s *d* = 2.09) and less controlled secondary performance. The standardized-model estimate included no difference (0.91 ± 0.06 vs. 0.87 ± 0.08; *p* = .082), whereas noncanonical-case accuracy was lower in the pure-VR condition (0.52 ± 0.14 vs. 0.71 ± 0.11; MD, − 0.19; 95% CI, − 0.27 to − 0.11). Immediate scores were compatible with no difference; at four weeks, the pure-VR condition scored lower (11.75 ± 4.63 vs. 17.20 ± 4.05; MD, − 5.45; 95% CI, − 8.24 to − 2.66). The reported condition-by-time interaction was *F*(2, 76) = 15.38, *p* < .001, partial η² = 0.29.

**Conclusions:**

Pure-VR instruction showed no clear deficit in immediate learning or standardized recognition, but was associated with less controlled tissue handling, lower transfer to noncanonical images, and weaker delayed retrieval. Because condition was confounded with intact class, the sample was small, and the modules were analyzed post hoc, the findings are exploratory and noncausal. VR may be most useful within a competency-aligned blended curriculum.

**Supplementary Information:**

The online version contains supplementary material available at https://doi.org/10.1186/s12909-026-10481-7.

## Background

Virtual reality (VR), augmented reality, and interactive three-dimensional resources are increasingly used in health-professions anatomy education. These technologies can make spatial relationships visible, allow repeated manipulation from multiple viewpoints, and broaden access when cadaveric material, laboratory time, or specialist teaching is constrained. Systematic reviews and meta-analyses generally report benefits for engagement, spatial understanding, and selected knowledge outcomes, while also identifying substantial heterogeneity across anatomical content, comparators, exposure duration, devices, and assessment methods [1–6]. A central limitation of this evidence is that immediate structure identification on a familiar representation does not establish transfer to other functions of anatomical knowledge.

Educational modalities are better evaluated according to the information and practice they provide than as interchangeable competitors. Digital anatomy offers repeatability, segmentation, learner-controlled viewpoints, and safe rehearsal [7]. Cadaver-based learning provides physical scale, texture, spatial constraint, tissue resistance, and exposure to biological variability. Comparative studies suggest that physical or cadaveric resources can be advantageous for some forms of relational or transfer knowledge, whereas immersive VR can produce comparable or better outcomes for selected factual and engagement measures [8–13]. These findings support functional complementarity: each modality may be effective for particular objectives without reproducing the full learning environment of the other.

Haptic information is especially relevant when anatomy must guide tissue handling. During dissection, learners integrate vision with resistance, deformation, texture, instrument position, and the consequences of force. Many commercial VR anatomy systems provide visual and auditory feedback but no tissue-level force feedback, and even dedicated haptic simulators approximate only selected mechanical properties [14]. Embodied-cognition accounts predict that changing sensorimotor contingencies can alter the representations formed during learning and their subsequent transfer [15]. Despite this theoretical relevance, evaluations of VR anatomy education rarely include standardized performance on real tissue.

Transfer across representations is another unresolved issue. Standardized three-dimensional models are valuable for establishing canonical relationships, but clinical images and cadaveric specimens contain anatomical variation, pathology, distortion, and ambiguity. A learner may recognize an idealized model accurately yet struggle when surface form or imaging context changes. Because standardized VR models and CT/MRI cases also differ in medium, a comparison between them cannot isolate biological variability alone; it can, however, test transfer to noncanonical clinical representations.

The durability of learning is equally important. VR can reduce the effort required to rotate, isolate, label, and retrieve a structure. Such support may reduce extraneous demands during initial learning, but immediate fluency is not synonymous with durable access. Research on desirable difficulties suggests that effortful retrieval and internally generated relations can strengthen later performance [16]. Work on cognitive offloading provides a reason to investigate how external access changes encoding, although it does not establish that digital tools necessarily weaken memory [17]. Reviews of immersive anatomy education have therefore called for delayed assessment and outcomes beyond factual recall [1, 18].

We integrated data from three routine educational-assessment modules comparing pure-VR and cadaver-based instruction: haptic transfer to a cadaver task, recognition of standardized and noncanonical clinical anatomy, and knowledge retention over four weeks, with the analysis specified post hoc. We examined whether the teaching conditions were associated with differences in tissue handling, transfer across representations, and delayed retrieval. The purpose was not to determine whether VR is globally superior or inferior, but to identify boundary conditions relevant to competency-aligned curriculum design.

## Methods

### Study design, setting, and reporting

This was a single-center retrospective multimethod cohort comparison based on routine educational-assessment data collected at Hubei University of Medicine, China, from September 2025 through January 2026. Three modules combined performance-based assessment, representation transfer, and repeated knowledge testing. The same two intact first-year classes participated across modules. Each module was originally conducted as a routine assessment in a different anatomical region; the data were collected prospectively during routine teaching, and the decision to combine the three modules into one comparative analysis was made post hoc. The report was guided by relevant items from the Strengthening the Reporting of Observational Studies in Epidemiology (STROBE) statement [19].

The modules addressed distinct learning functions: forearm anatomy for haptic transfer, standardized models and CT/MRI cases for representation transfer, and cranial-base foramina for delayed retention. Outcomes were not combined into a composite score. Three focal module outcomes were designated post hoc for the integrated analysis: peak manipulation force, accuracy for noncanonical clinical cases, and the condition-by-time interaction in knowledge scores. Other outcomes were considered secondary or exploratory. The term *focal* distinguishes this retrospective designation from prospective outcome prespecification.

### Participants, allocation, and participant flow

Forty first-year clinical-medicine students without previous cadaver-dissection experience were included. All participants were admitted directly from high school and had no prior university-level laboratory coursework involving animal dissection or tissue manipulation. However, as is standard in Chinese secondary education, all students had completed basic high-school biology courses that typically include simple animal dissection (e.g., frogs or mice). Such experience was universal across all participants and therefore could not account for between-group differences in the haptic-transfer assessment.

Under the existing curriculum, one intact class received pure-VR instruction and one intact class received cadaver-based instruction (20 students per class). There was no sequence generation, allocation concealment, or researcher-controlled assignment. Teaching condition was therefore fully confounded with class, and the same class-level allocation was retained across the three modules.

The two intact classes were formed by the university’s academic registry based solely on student identification numbers at the time of enrollment. This administrative procedure did not consider academic performance, entrance examination scores, or student preferences, and thus approximated a natural randomization at the class level. To empirically confirm baseline comparability, we compared the two cohorts on measured characteristics (age, sex, and prior anatomy entrance scores). As shown in Table S2, all absolute standardized mean differences (SMDs) were below 0.22, indicating good balance between the two observed cohorts on these measured variables. All anatomy teaching and assessments were conducted in Chinese (Mandarin), which is the language of instruction at the institution. The VR software interface (3D Organon XR) displayed English anatomical labels with Chinese subtitles, and the instructor provided oral guidance in Chinese during all VR sessions. The three modules were scheduled at Weeks 4, 8, and 12 of the same semester, each following a common lecture (2 h) delivered to both classes together. Although we cannot rule out unmeasured confounding, these data suggest that the class-formation process did not introduce a severe systematic selection bias.

Eligibility required enrollment in the relevant anatomy course, no previous cadaver-dissection experience, and consent for secondary research use of anonymized routine educational-assessment data. Teaching records indicated no exclusions, withdrawals, or missing assessments. All 40 students were included at each scheduled time point. No prospective power calculation was performed because the sample comprised the available teaching cohorts. Interpretation consequently emphasizes estimation and uncertainty and treats the comparison as exploratory.

### Learning conditions

The three modules were selected components embedded within a broader, semester-long anatomy course that also covered other regions such as the thorax, abdomen, and lower limbs. Table 1 provides a chronological summary of the three modules, including anatomical content, learning objectives, duration, instructional activities for each condition, instructor ratio, device/specimen availability, estimated hands-on time per student, and common supporting materials received before each module.

**Table 1:** Chronological summary of the three instructional modules

Item	Module 1: Haptic transfer	Module 2: Representation transfer	Module 3: Retention
Anatomical region	Forearm flexor region	Abdominal and pelvic vascular structures	Cranial-base foramina
Learning objectives	Identify and mobilize radial artery; develop instrument handling	Recognize structures on standardized models and cross-sectional imaging	Identify foramina and their traversing nerves/vessels
Order and timing	Week 4 of semester	Week 8 of semester	Week 12 of semester
Duration of instruction	8 contact hours	6 contact hours	6 contact hours
VR group activities	3D Organon XR visualization, structure isolation, label-based identification	Same VR platform with standardized 3D models	VR exploration of cranial base, foramina isolation, nerve/vessel tracing
Cadaver group activities	Dissection and identification on formalin-fixed specimens	Specimen-based identification with cross-sectional correlation	Isolated skulls and dissected specimens with nerve/vessel tracing
Common lectures before module	2 h lecture on forearm anatomy	2 h lecture on abdominal/pelvic anatomy + 2 h radiology introduction	2 h lecture on cranial anatomy
Prior modality exposure before module	None (first hands-on session)	VR group: 8 h prior VR exposure; Cadaver group: 8 h prior cadaver exposure	VR group: 14 h prior VR exposure; Cadaver group: 14 h prior cadaver exposure
Instructor-to-student ratio	1:10	1:10	1:10
VR devices	10 headsets (2 students per device)	10 headsets (2 students per device)	10 headsets (2 students per device)
Approx. individual hands-on time	~ 2 h (VR) / ~3 h (cadaver)	~ 1.5 h (VR) / ~2.5 h (cadaver)	~ 2 h (VR) / ~3 h (cadaver)
Cadaveric specimens	4 specimens (4–5 students per specimen)	4 specimens (4–5 students per specimen)	5 isolated skulls (4 students per skull) + 2 dissected specimens
Supporting materials	Same textbook, same lecture slides, same demonstration videos for both groups	Same textbook, same lecture slides, same imaging atlas for both groups	Same textbook, same lecture slides, same skull diagrams for both groups

All three modules were embedded within a broader semester-long anatomy course. Prior modality exposure is cumulative from previous modules. *h*   hours

For the haptic-transfer module, both cohorts studied the forearm flexor region for eight contact hours under the supervision of the same instructor. Before modality-specific practice, both groups received the same oral orientation to the relevant structures. The student-to-instructor ratio was approximately 10:1.

The pure-VR class used HTC VIVE Pro 2 head-mounted displays and handheld controllers (HTC Corporation, Taiwan) with 3D Organon XR (Medis Media, Australia). The software version was not retained in the routine teaching record. Interaction was limited to visual and auditory feedback; the system provided no tissue-level force feedback. The cadaver class studied the same region using formalin-fixed specimens and standard dissection instruments (scalpels, dissection scissors, and tissue forceps), with four to five students per specimen, under direct instructor guidance. Scheduled contact time and broad learning objectives were matched, but the physical activities and feedback available in the two conditions necessarily differed.

For the retention module, each cohort learned the cranial-base foramina and their traversing nerves and vessels using its assigned modality. Access to additional study resources during the follow-up interval was not restricted or measured, and both groups continued to receive anatomy teaching on other regions (thorax, abdomen, lower limbs) during the four-week retention period.

### Haptic-transfer assessment

One week after forearm instruction, each participant completed a standardized cadaveric task: exposure and mobilization of a 5-cm segment of the distal radial artery. FlexiForce A201 thin-film force sensors (Tekscan Inc., Norwood, MA, USA) attached to the handles of forceps and scissors recorded applied force. The archived protocol specified acquisition at 100 Hz through a Tekscan data-acquisition interface. A Sony PMW-10MD high-definition medical video camera (Sony Corporation, Japan) recorded hand and instrument movement. Sensors were calibrated before use according to the manufacturer’s instructions. Sensor serial numbers, the exact acquisition-interface model, and full signal-processing documentation were not retained.

Peak manipulation force was the focal module outcome. Secondary outcomes were trajectory-direction changes per second and overall manipulation quality on a five-point scale. Counts above 5 N and recorded injuries to critical structures were treated as exploratory. The historical rationale for choosing 5 N could not be reconstructed; it is therefore described as an operational threshold and is not interpreted as a validated clinical safety limit. Teaching condition was concealed from the outcome assessor. The available record did not contain sufficient detail to reproduce the movement algorithm, injury rubric, or all anchors of the five-point quality scale.

### Recognition of standardized and noncanonical anatomy

Participants viewed 20 representations in computer-generated random order. Set A comprised 10 standardized models from the same 3D Organon XR platform used by the pure-VR class. Set B comprised 10 de-identified CT (n = 6) and MRI (n = 4) cases from the same abdominal and pelvic vascular regions as Set A, retrieved from the institutional picture archiving and communication system. The CT images were axial, and the MRI images were coronal. The cases contained clearly visible anatomical variants (e.g., replaced hepatic artery, retroaortic left renal vein) or pathological alterations (e.g., aortic aneurysm, renal artery stenosis), as documented in the radiology report. A radiologist with 15 years of experience selected the cases and annotated the designated vascular target structures (e.g., celiac trunk branches, superior mesenteric artery, renal arteries), which corresponded anatomically to the structures depicted in Set A. Set A and Set B thus assessed the same anatomical content but differed in presentation medium (standardized 3D models versus clinical cross-sectional images). All target structures had been taught to both classes in common lectures prior to the module. The annotation is described as an expert reference rather than a gold standard because it was provided by one specialist. Each representation was displayed for 30 seconds, after which participants identified the designated structure. Responses were classified into three mutually exclusive categories: (1) accuracy — the participant correctly named the designated structure; (2) misjudgment — the participant named an incorrect structure (i.e., a different structure from the target); and (3) omission — the participant either provided no response or explicitly stated ‘uncertain’ or ‘cannot identify’ within the 30-second display period. Rates for each category were calculated using the total number of items per set (10 items) as the denominator for each participant. Misjudgment is not simply the complement of accuracy; it specifically captures active errors of commission, whereas omissions represent failure to respond or uncertainty. This distinction is educationally meaningful because active misidentification suggests a different learning gap than lack of response. Scorers were unaware of teaching condition. Accuracy for Set B was the focal module outcome. Set A accuracy and the rate of misjudgment in Set B were secondary outcomes. Set A also matched the pure-VR class’s learning platform, whereas Set B differed in biological content and presentation medium. The analysis was therefore interpreted as transfer to noncanonical clinical representations, not as an isolated effect of anatomical variation or a modality-neutral comparison of the two item sets. Institutional authorization covered educational research use of the de-identified images.

### Knowledge-retention assessment

Knowledge of cranial-base foramina and traversing structures was assessed 30 min after instruction (T0), after one week (T1), and after four weeks (T2). During each closed-book assessment, participants used an unlabeled skull model to identify the cribriform foramina, optic canal, superior orbital fissure, foramen rotundum, foramen ovale, foramen spinosum, jugular foramen, hypoglossal canal, internal acoustic meatus, and foramen magnum. Correct identification of each foramen received 1 point, and correct identification of its major traversing nerve or vessel received 2 additional points (maximum score, 30). The same assessment form was used at each time point, and participants were not informed in advance that delayed tests would occur.

Two anatomy educators developed the assessment and piloted its scoring with 10 students who did not enter the main comparison. The teaching record reported an inter-rater correlation above 0.90, but the statistic used and its confidence interval were not retained. The condition-by-time interaction was the focal module outcome. Time-specific between-condition contrasts and ratios of group means relative to T0 were used for interpretation. The latter are descriptive and are not equivalent to the mean of participant-level retention ratios.

### Statistical analysis

Continuous variables are summarized as means and standard deviations. Baseline differences are described using absolute standardized mean differences (SMDs); no baseline significance tests were used. The archived analysis used independent-samples *t* tests for continuous outcomes and Mann–Whitney *U* tests for markedly non-normal or ordinal outcomes. Mean differences (MDs), 95% confidence intervals (CIs), and Cohen’s *d* are reported for continuous comparisons. Complete distributional diagnostic output was not retained.

Knowledge scores were analyzed using a 2-condition × 3-time repeated-measures analysis of variance. Greenhouse–Geisser correction was planned when sphericity was not supported. The condition-by-time interaction was the focal retention test and is reported with partial eta squared (partial η²). Time-specific comparisons were secondary. As a conservative sensitivity rule for the three post hoc focal tests, statistical interpretation used a Bonferroni-adjusted threshold of 0.0167; unadjusted two-sided *p* values are displayed. This adjustment does not convert retrospective outcome selection into prospective prespecification. Secondary and exploratory outcomes were not multiplicity-adjusted and are interpreted accordingly. No outcome data were missing. Analyses were reported as conducted in SPSS version 26.0.

Because one intact class represented each teaching condition, class-level variation could not be separated from condition. The individual-level CIs and *p* values quantify variation among students within these two classes but do not incorporate between-class uncertainty; they may therefore overstate precision for a modality effect. All inferential results are interpreted as exploratory associations within the observed cohorts.

The source statistical table for injury counts reported a Mann–Whitney result of *p* < .001 but did not retain the *U* statistic. Because exact output could not be verified, injury counts are presented descriptively without an inferential *p* value. This decision does not affect any focal module outcome.

### Exploratory analyses reported separately

One exploratory evaluation is retained in Additional file 1 for descriptive transparency and does not contribute to the principal conclusions: a nonrandomized senior mixed-learning benchmark for the representation task. The senior cohort was not comparable to the first-year cohorts in academic stage, cumulative anatomy exposure, or clinical-imaging experience. This benchmark is therefore presented descriptively and does not support causal or comparative inferences about mixed-learning modalities.

### Ethics approval and consent

This study was conducted in accordance with the Declaration of Helsinki. The data arose from routine anatomy teaching and anonymized educational assessments. VR and cadaver instruction were components of the regular curriculum rather than clinical interventions. The study protocol, including secondary research use of the data, was approved by the Ethics Review Committee of Hubei University of Medicine on 19 July 2025 (approval no. 2025-RE-024), before the study period. The approval covered retrospective analysis of routine educational data, including cadaveric assessment, sensor and video recording, review of de-identified clinical images, repeated knowledge testing, and verbal-protocol recordings. Students provided written informed consent at the beginning of the academic year for research use of anonymized data generated during routine teaching.

### Use of generative AI in manuscript preparation

A generative AI-based language tool was used during manuscript revision for English-language editing, organization, and grammar checking. It was not used to generate or alter study data, perform statistical analyses, or determine the scientific conclusions. The authors reviewed and take responsibility for all text, citations, analyses, and interpretations.

## Results

### Participants and baseline characteristics

All 40 first-year students completed the three modules, with 20 participants analyzed per condition at every time point. No exclusions, withdrawals, or missing assessments were recorded.

Measured age, sex distribution, and baseline anatomy score differed modestly between classes (Table S2); absolute SMDs ranged from 0.10 to 0.21.

These measured characteristics do not exclude imbalance in motivation, spatial ability, previous digital-learning experience, or other class-level factors.

### Haptic transfer to cadaveric tissue

Peak manipulation force was higher in the pure-VR condition than in the cadaver condition (8.74 ± 2.31 vs. 4.52 ± 1.68 N; MD, 4.22 N; 95% CI, 2.92–5.52; *p* < .001; *d* = 2.09) (Table 2). This focal comparison remained below the multiplicity-adjusted threshold.

**Table 2:** Haptic-transfer and manipulation-performance outcomes

Outcome	Pure-VR (*n* = 20)	Cadaver (*n* = 20)	Mean difference (95% CI)	*p*	Effect size
Peak manipulation force, N†	8.74 ± 2.31	4.52 ± 1.68	4.22 (2.92 to 5.52)	< 0.001	*d* = 2.09
Direction changes, /s	2.87 ± 0.64	1.32 ± 0.41	1.55 (1.20 to 1.90)	< 0.001	*d* = 2.88
Manipulation quality, 1–5	2.25 ± 0.72	3.90 ± 0.55	−1.65 (− 2.06 to − 1.24)	< 0.001	*d* = − 2.58
Crossings of operational 5-N threshold, *n*‡	7.85 ± 3.12	2.15 ± 1.79	5.70 (4.06 to 7.34)	< 0.001	*d* = 2.24
Recorded critical-structure injuries, *n*‡	1.65 ± 0.81	0.45 ± 0.60	Not estimated	—	Mean ratio = 3.67

Mean difference = pure-VR minus cadaver. †Post hoc focal module outcome. ‡Exploratory outcome. The 5-*N* value is an operational threshold, not a validated clinical safety limit

Secondary performance measures showed a consistent direction. The pure-VR condition changed direction more frequently (MD, 1.55 changes/s; 95% CI, 1.20–1.90) and received lower manipulation-quality ratings (MD, − 1.65 points; 95% CI, − 2.06 to − 1.24). It also crossed the exploratory 5-N threshold more often. Recorded injuries averaged 1.65 ± 0.81 in the pure-VR condition and 0.45 ± 0.60 in the cadaver condition; because exact count-model or Mann–Whitney output was unavailable, no inferential estimate is attached to this exploratory measure. These outcomes concern performance on a formalin-fixed cadaver task and are not evidence of patient harm.

### Recognition of standardized and noncanonical anatomy

Accuracy was high in both conditions for standardized models, and the between-condition estimate included no difference (0.91 ± 0.06 vs. 0.87 ± 0.08; MD, 0.04; 95% CI, − 0.005 to 0.085; *p* = .082) (Table 3). For noncanonical CT/MRI cases, accuracy was lower in the pure-VR condition (0.52 ± 0.14 vs. 0.71 ± 0.11; MD, − 0.19; 95% CI, − 0.27 to − 0.11; *p* < .001; *d* = − 1.51). This focal comparison remained below the multiplicity-adjusted threshold. The pure-VR condition also had a higher secondary misjudgment rate (MD, 0.20; 95% CI, 0.13–0.27).

**Table 3:** Recognition of standardized models and noncanonical clinical images

Outcome	Pure-VR (*n* = 20)	Cadaver (*n* = 20)	Mean difference (95% CI)	*p*	Effect size
Standardized-model accuracy	0.91 ± 0.06	0.87 ± 0.08	0.04 (− 0.005 to 0.085)	0.082	*d* = 0.57
Noncanonical-case accuracy†	0.52 ± 0.14	0.71 ± 0.11	−0.19 (− 0.27 to − 0.11)	< 0.001	*d* = − 1.51
Noncanonical-case misjudgment rate	0.38 ± 0.13	0.18 ± 0.10	0.20 (0.13 to 0.27)	< 0.001	*d* = 1.72

Mean difference = pure-VR minus cadaver. †Post hoc focal module outcome

Because the two sets differed in content and presentation medium, and Set A matched the pure-VR learning platform, the results show a condition-associated difference in transfer to noncanonical clinical images; they do not isolate a causal effect of anatomical variation, pathology, or CT/MRI interpretation. The remaining responses not accounted for by accuracy and misjudgment were classified as omissions (no response or ‘uncertain’). The omission rates were 10% in the pure-VR class (100% − 52% − 38%) and 11% in the cadaver class (100% − 71% − 18%).

### Immediate and delayed knowledge retention

At T0, the pure-VR group scored 1.75 points higher on average, but the CI included no difference (95% CI, − 0.69 to 4.19; *p* = .154). At T1, the estimate also remained compatible with no group difference (MD, − 1.45; 95% CI, − 4.04 to 1.14; *p* = .265). At T2, the pure-VR group scored 5.45 points lower (95% CI, − 8.24 to − 2.66; *p* < .001; *d* = − 1.25) (Table 4).

**Table 4:** Knowledge scores and retention over four weeks

Time point	Pure-VR score	Cadaver score	Mean difference (95% CI)	*p*	Ratio of group mean to T0, VR/cadaver
T0: 30 min	24.85 ± 3.42	23.10 ± 4.15	1.75 (− 0.69 to 4.19)	0.154	1.000 / 1.000
T1: 1 week	18.40 ± 4.21	19.85 ± 3.88	−1.45 (− 4.04 to 1.14)	0.265	0.741 / 0.859
T2: 4 weeks	11.75 ± 4.63	17.20 ± 4.05	−5.45 (− 8.24 to − 2.66)	< 0.001	0.473 / 0.745

Maximum score = 30. Mean difference = pure-VR minus cadaver. Time-specific contrasts are secondary; the condition-by-time interaction was the post hoc focal module test

The reported condition-by-time interaction was *F*(2, 76) = 15.38, *p* < .001, partial η² = 0.29, indicating different score trajectories within the archived individual-level analysis. This focal comparison remained below the multiplicity-adjusted threshold. At four weeks, the ratio of each condition’s mean score to its T0 mean was 47.3% for pure VR and 74.5% for cadaver-based instruction. These ratios are descriptive.

## Discussion

### Principal findings

Across three educational functions, pure-VR instruction showed a differentiated pattern rather than uniform inferiority or superiority. Immediate knowledge scores and standardized-model recognition were compatible with no between-condition difference. By contrast, the pure-VR class used greater peak force in a subsequent cadaver task, performed less well on noncanonical clinical images, and had a steeper decline in knowledge scores over four weeks. This pattern is consistent with visual immersion supporting canonical visualization and immediate learning while providing less opportunity to practice sensorimotor calibration, transfer across representations, and delayed unaided retrieval.

These conclusions are intentionally associative. Each teaching condition was represented by only one intact class, so modality and class were inseparable. The same instructor, similar contact time, and modest measured baseline differences reduce some alternative explanations but do not address unmeasured class-level, motivational, or exposure-related confounding. Individual-level uncertainty estimates also omit between-class variation. The results identify hypotheses and curriculum-relevant boundary conditions; they do not establish that VR itself caused the observed differences.

Our findings of comparable immediate learning but weaker delayed retention and transfer are not uniformly supported by the existing literature. Several randomized trials and meta-analyses have reported that immersive VR can produce learning outcomes equivalent to or even superior to traditional methods, particularly for factual knowledge and spatial understanding [6, 10, 11]. For example, Kurul et al. found that VR-based neuroanatomy training yielded test scores comparable to cadaver-based instruction at immediate post-test [11], and Zhao et al.‘s meta-analysis concluded that VR generally improves anatomy knowledge scores relative to non-VR controls [6]. However, these studies predominantly assessed immediate factual recall using standardized representations, with few including delayed testing or transfer to noncanonical materials. Our study extends this evidence by identifying specific conditions—haptic transfer, cross-representational recognition, and long-term retrieval—under which VR without force feedback may be less effective. This differentiated pattern is consistent with the view that modality effectiveness depends on the targeted learning outcome, rather than being an inherent property of the technology itself.

### Haptic performance and practice specificity

The largest estimated differences occurred in the cadaver transfer task. Cadaver-taught students had already practiced tissue resistance, deformation, instrument constraints, and the consequences of force. The pure-VR system provided none of these cues. The most defensible explanation is therefore practice specificity: sensorimotor information unavailable during learning did not transfer automatically to physical tissue handling. This account is consistent with embodied approaches to learning and the role of haptic information in procedural simulation [14, 15].

The assessment also favored physical similarity between cadaver instruction and cadaver testing. This transfer-appropriate advantage is educationally relevant when tissue handling is an intended competency, but it prevents separation of cadaver exposure from manual practice, contextual similarity, and instructor feedback. A future factorial study should compare visual VR, VR with validated force feedback, cadaver observation, active dissection, and sequenced blended instruction while matching practice time and feedback.

The force and movement findings must remain confined to the measured task. Formalin-fixed tissue differs mechanically from living tissue; the exact acquisition interface and signal-processing details were not retained; the basis of the 5-N operational threshold could not be reconstructed; and no clinical outcome was measured. Accordingly, the results support further testing of direct haptic practice when a curriculum assesses tissue handling, but not a claim about patient safety.

### Transfer to noncanonical clinical representations

The recognition module distinguished performance on standardized models from transfer to CT/MRI cases containing variation or pathology. Set A came from the same platform used by the pure-VR class and therefore favored representational familiarity for that condition. The absence of a clear Set A difference is compatible with pure VR supporting canonical recognition. Lower accuracy on noncanonical cases may indicate narrower representation experience, but the design does not isolate biological variability. Set B also differed from Set A in imaging medium, visual complexity, and pathological context. The outcome is therefore best understood as transfer to noncanonical clinical representations.

This limitation points directly to an instructional opportunity. Digital anatomy is not intrinsically restricted to one idealized model. A variation-enriched platform could incorporate multiple specimens, cross-sectional imaging, pathology, uncertainty prompts, and deliberate comparison across representations. Future same-stage randomized studies should test standardized-only VR against variation-enriched VR, cadaveric learning, and blended sequences, with participant-by-item mixed-effects models and more than one expert reference assessor.

### Delayed retention

The retention trajectory illustrates why immediate assessment alone can be misleading. Initial scores were compatible with no group difference, while the four-week estimate favored the cadaver cohort. Effortful retrieval, contextual elaboration, and internally reconstructed spatial relations are plausible explanations [16], but they were not measured. Cognitive-offloading theory likewise motivates investigation of encoding behavior [17], not a conclusion that VR caused “memory outsourcing.”

Unmeasured independent study, access to additional resources, repeated use of the same assessment form, and baseline cognitive differences could also explain part of the trajectory. The two intact classes may have differed in unmeasured variables such as independent study habits, motivation, peer-learning engagement, examination-preparation strategies, or use of digital resources. Such class-level differences, rather than the instructional modality alone, could account for part or all of the observed divergence in four-week scores. The repeated use of the same assessment form may also have introduced differential testing or retrieval-practice effects between groups. Therefore, the proposed mechanisms involving effortful retrieval, contextual elaboration, and cognitive offloading should be interpreted as untested hypotheses requiring direct measurement in future studies, not as demonstrated explanations for the observed retention difference.

Future studies should use validated parallel forms, monitor interval study, prespecify the principal delayed time point, and analyze participant-level trajectories using mixed-effects models. The present ratio-of-group-means is intuitive but should not be mistaken for participant-level retention.

### Implications for curriculum design

The practical implication is alignment between modality and competency. VR is well suited to orientation, repeated visualization, segmentation, and learner-controlled exploration. When objectives include tissue discrimination, instrument control, or the consequences of force, direct cadaveric or validated haptic practice is more authentic. When objectives include robust recognition across biological and clinical variability, learners need multiple specimens and imaging modalities. When durable access is required, instruction should include retrieval without labels and delayed unaided assessment.

A defensible blended sequence would use VR for preparatory orientation, require unlabeled reconstruction and retrieval, introduce multiple variant and pathological cases, add cadaveric or validated haptic practice for relevant competencies, and use structured reflection to connect physical observations with imaging and clinical mechanisms. The present data do not establish an optimal proportion of VR or cadaver time; dosage should be tested against explicitly defined outcomes.

### Strengths and limitations

The study extends beyond immediate factual tests by examining performance on tissue, transfer to noncanonical images, and four-week retention in the same teaching cohorts. Reporting mean differences, confidence intervals, and effect sizes improves interpretability, and restricting the main analysis to same-stage cohorts avoids the most serious confounding introduced by the senior benchmark.

Several limitations constrain inference. Most importantly, the study had one intact class per condition; modality effects cannot be separated from class effects, and individual-level tests may overstate precision. A crossover or sequential design would have ensured that all students received equal exposure to both VR and cadaver-based instruction, addressing both educational equity and methodological rigor. However, such a design was not feasible in this real-curriculum setting because the VR module was scheduled as a fixed component for only one intact class during the study period, and the course timetable did not permit swapping modalities mid-semester. We acknowledge this as an ethical and practical constraint. Future prospective studies should consider crossover, cluster-randomized, or sequential designs to ensure equitable access to both instructional modalities while isolating modality-specific effects. The study was also nonrandomized, single-center, small, and not prospectively powered. The three modules and focal outcomes were analyzed post hoc, increasing the risk of selective emphasis. Participants and instructors could not be blinded. The cadaver class had greater physical similarity between instruction and the transfer test, whereas the pure-VR class had greater representational similarity between instruction and Set A. Secondary outcomes were not multiplicity-adjusted. The software version, exact sensor-acquisition interface, signal-processing details, complete manipulation rubrics, and precise reliability statistic for the retention assessment were not retained. A single radiologist supplied the expert reference for the clinical images. The same retention form was repeated, interval study was not controlled, and archived diagnostic output was insufficient to confirm every repeated-measures assumption independently. No clinical outcomes were measured. These limitations make the study hypothesis-generating rather than definitive.

## Conclusions

In this retrospective comparison of two intact classes, pure-VR anatomy instruction showed no clear deficit in immediate learning or standardized recognition, but was associated with less controlled cadaver-task performance, lower recognition of noncanonical clinical images, and weaker four-week retention. These observations identify testable limits of visually immersive instruction without tissue-level force feedback; they do not show that VR is generally ineffective or establish causal effects. Competency-aligned blended curricula may preserve the functions that VR supports while adding direct practice when objectives require tissue interaction, transfer across biological representations, or durable unaided retrieval.

## Supplementary Information

Supplementary Material 1.

## Data Availability

No datasets were generated or analysed during the current study.

## Abbreviations

CI: Confidence interval
CT: Computed tomography
MD: Mean difference
MRI: Magnetic resonance imaging
SMD: Standardized mean difference
STROBE: Strengthening the reporting of observational studies in epidemiology
VR: Virtual reality
