# Supplementary material for: Haptic and cognitive trade-offs of virtual reality–supported anatomy education: a retrospective multimethod cohort comparison
Source: BMC Med Educ. 2026 Sep 25;26:1595. doi: 10.1186/s12909-026-10481-7 (PMC13644259; doi:10.1186/s12909-026-10481-7)
Supplement: Supplementary file 1 [file 12909_2026_10481_MOESM1_ESM.docx]

# **Additional file 1**

## **Exploratory Analyses Excluded From the Principal Inferential Narrative**

**Related manuscript:** *Haptic and Cognitive Trade-offs of Virtual Reality–Supported Anatomy Education: A Retrospective Multimethod Cohort Comparison*

**Authors:** Songyuan Yao, Rui Liu, and Xiju He

This file reports two analyses retained for transparency but excluded from the main abstract and principal conclusions. Both are hypothesis-generating. The first uses a noncomparable senior benchmark; the second uses a small subsample and compositional verbal-protocol data with incomplete AI-system metadata.

# **S1. Senior mixed-learning benchmark in the representation task**

## **Methods**

Twenty senior medical students who had previously learned anatomy using both digital resources and cadaver-based instruction completed the same 20-item representation task as the two first-year cohorts. They viewed 10 standardized models (Set A) and 10 de-identified CT/MRI cases containing an anatomical variant or pathological alteration (Set B). The presentation order was computer-randomized, and each item was shown for 30 seconds.

The senior students differed from the primary cohorts in academic stage, cumulative anatomy exposure, clinical-imaging experience, and duration of learning. Their results were therefore summarized descriptively and were not included in the main inferential comparisons. No claim about the effect of blended learning is made.

## **Results**

The senior mixed-learning group achieved standardized-model accuracy of 0.93 ± 0.05, noncanonical-case accuracy of 0.78 ± 0.09, and a noncanonical-case misjudgment rate of 0.15 ± 0.08 (Table S1). The values are shown only to contextualize the range observed in a more experienced cohort.

**Table S1. Descriptive representation-task outcomes across all teaching cohorts**

| **Outcome** | **Pure-VR first-year cohort (*n* = 20)** | **Cadaver first-year cohort (*n* = 20)** | **Senior mixed-learning benchmark (*n* = 20)** |
| --- | --- | --- | --- |
| Standardized-model accuracy | 0.91 ± 0.06 | 0.87 ± 0.08 | 0.93 ± 0.05 |
| Noncanonical-case accuracy | 0.52 ± 0.14 | 0.71 ± 0.11 | 0.78 ± 0.09 |
| Noncanonical-case misjudgment rate | 0.38 ± 0.13 | 0.18 ± 0.10 | 0.15 ± 0.08 |

*Note. Values are mean ± standard deviation. The senior benchmark was neither randomized nor matched and is not a causal comparator.*

## **Interpretation**

The senior cohort’s higher descriptive performance on noncanonical cases could reflect additional coursework, repeated exposure, clinical-imaging experience, cadaveric learning, digital learning, or other unmeasured differences. It cannot be attributed to a mixed-learning modality. A valid test of blended instruction would require same-stage learners allocated to clearly defined conditions with equivalent exposure and participant-by-item analysis.

# **S2. Exploratory think-aloud substudy with optional AI assistance**

## **Methods**

Ten students from each first-year teaching condition completed a standardized reasoning task based on a Pancoast-tumor presentation that included hoarseness, medial upper-limb pain, miosis, and ptosis. Participants were asked to verbalize their thinking and could use the same AI assistant as an optional information source. The interface and system prompt were held constant, and conversation history was cleared before each session. The specific AI product version and access date were not retained in the routine teaching record, which materially limits reproducibility.

If a participant remained silent for more than 30 seconds, the researcher used a neutral prompt to resume verbalization. The procedure followed established principles for verbal-protocol analysis [1]. Recordings were transcribed verbatim and segmented into independent clauses.

Two coders independently coded the transcripts. They first coded 20% of the material, discussed disagreements, refined the category definitions, and then completed the dataset. Categories were retrieval-oriented questions, hypothesis-driven questions, logical connective statements, AI-dependent statements, and critique or correction. Each participant’s category count was divided by that participant’s total coded clauses, so the percentages were compositional and summed to approximately 100%. Inter-coder agreement was summarized using Cohen’s kappa [2].

Because the subsample contained only 10 participants per group and the code proportions were compositional, the results were reported descriptively. The task did not measure diagnostic accuracy and cannot establish cognitive dependence.

## **Results**

Inter-coder agreement was reported as κ = .84. The pure-VR condition completed the task more quickly (8.40 ± 2.33 vs 12.11 ± 3.13 minutes). Retrieval-oriented questions and AI-dependent statements represented 71.6% of coded clauses in the pure-VR condition and 25.4% in the cadaver condition. Hypothesis-driven questions, logical connective statements, and critique or correction represented 28.5% and 74.7%, respectively; small discrepancies from 100% reflect rounding (Table S2).

**Table S2. Think-aloud task characteristics and code proportions**

| **Outcome** | **Pure-VR cohort (*n* = 10)** | **Cadaver cohort (*n* = 10)** | **Difference (VR − cadaver)** |
| --- | --- | --- | --- |
| Task duration, min | 8.40 ± 2.33 | 12.11 ± 3.13 | −3.71 |
| Retrieval-oriented questions, % | 38.55 ± 8.22 | 11.55 ± 4.72 | 27.00 |
| AI-dependent statements, % | 33.04 ± 7.56 | 13.86 ± 5.16 | 19.18 |
| Hypothesis-driven questions, % | 10.11 ± 4.33 | 26.21 ± 6.13 | −16.10 |
| Logical connective statements, % | 11.95 ± 5.02 | 29.24 ± 7.32 | −17.29 |
| Critique and correction, % | 6.44 ± 3.26 | 19.23 ± 5.86 | −12.79 |

*Note. Values are mean ± standard deviation. Code proportions were calculated within participant. No inferential tests were performed.*

## **Interpretation and limitations**

The descriptive pattern may indicate differences in observable verbal behavior, but it does not show that one cohort had better clinical reasoning or that VR caused AI reliance. Plausible alternative explanations include prior knowledge, confidence, digital fluency, familiarity with the AI interface, task pacing, and willingness to verbalize. Think-aloud reports are incomplete traces of cognition, and the term *AI-dependent statement* describes a coding category rather than a stable learner characteristic.

Future studies should prospectively specify the task and coding manual, preserve model/version metadata and prompts, measure answer accuracy and confidence, record prompt-level behavior, and assess subsequent unaided transfer. Participant-level compositional models or count models would be preferable to independent tests of percentages that necessarily sum to 100%.

# **References for Additional file 1**

[1]Ericsson KA, Simon HA. *Protocol Analysis: Verbal Reports as Data.* Revised ed. Cambridge, MA: MIT Press; 1993.

[2]Cohen J. A coefficient of agreement for nominal scales. *Educ Psychol Meas.* 1960;20(1):37–46. doi:10.1177/001316446002000104.
